# Epidemiological Investigation of Bovine Tuberculosis Herd Breakdowns in Spain 2009/2011

**DOI:** 10.1371/journal.pone.0104383

**Published:** 2014-08-15

**Authors:** Sintayehu Guta, Jordi Casal, Sebastian Napp, Jose Luis Saez, Ariadna Garcia-Saenz, Bernat Perez de Val, Beatriz Romero, Julio Alvarez, Alberto Allepuz

**Affiliations:** 1 National Animal Health Diagnostic and Investigation Center (NAHDIC), Sebeta, Ethiopia; 2 Centre de Recerca en Sanitat Animal (CReSA), UAB-IRTA, Campus de la Universitat Autònoma de Barcelona, Bellaterra, Barcelona, Spain; 3 Departament de Sanitat i Anatomia Animals, Universitat Autònoma de Barcelona, Bellaterra, Barcelona, Spain; 4 Subdirección General de Sanidad e Higiene Animal y Trazabilidad, Dirección General de la Producción Agraria, Ministerio de Agricultura, Alimentación y Medio Ambiente, Madrid, Spain; 5 Centro de Vigilancia Sanitaria Veterinaria (VISAVET), Universidad Complutense de Madrid, Madrid, Spain; 6 Department of Veterinary Population Medicine, University of Minnesota, St Paul, Minnesota, United States of America; INIAV, I.P.- National Institute of Agriculture and Veterinary Research, Portugal

## Abstract

We analyzed the most likely cause of 687 bovine tuberculosis (bTB) breakdowns detected in Spain between 2009 and 2011 (i.e., 22% of the total number of breakdowns detected during this period). Seven possible causes were considered: i) residual infection; ii) introduction of infected cattle from other herds; iii) sharing of pastures with infected herds; iv) contiguous spread from infected neighbor herds; v) presence of infected goats in the farm; vi) interaction with wildlife reservoirs and vii) contact with an infected human. For each possible cause a decision tree was developed and key questions were included in each of them. Answers to these key questions lead to different events within each decision tree. In order to assess the likelihood of occurrence of the different events a qualitative risk assessment approach was used. For this purpose, an expert opinion workshop was organized and ordinal values, ranging from 0 to 9 (i.e., null to very high likelihood of occurrence) were assigned. The analysis identified residual infection as the most frequent cause of bTB breakdowns (22.3%; 95%CI: 19.4–25.6), followed by interaction with wildlife reservoirs (13.1%; 95%CI: 10.8–15.8). The introduction of infected cattle, sharing of pastures and contiguous spread from infected neighbour herds were also identified as relevant causes. In 41.6% (95%CI: 38.0–45.4) of the breakdowns the origin of infection remained unknown.

Veterinary officers conducting bTB breakdown investigations have to state their opinion about the possible cause of each breakdown. Comparison between the results of our analysis and the opinion from veterinary officers revealed a slight concordance. This slight agreement might reflect a lack of harmonized criteria to assess the most likely cause of bTB breakdowns as well as different perceptions about the importance of the possible causes. This is especially relevant in the case of the role of wildlife reservoirs.

## Introduction

Bovine tuberculosis (bTB) is a chronic infectious disease of cattle (including all *Bos* species); buffaloes (*Bubalus bubalis*) and bison (*Bison bison*) caused by any of the disease-causing mycobacterial species within the *Mycobacterium tuberculosis*-complex [1]. In industrialized countries, bTB control programs are mainly based on routine intra-dermal skin tests and removal of positive reactors supplemented by slaughterhouse surveillance [2]. In Europe, some countries have achieved the officially tuberculosis free (OTF) status, which implies reporting 99.9% of bTB-free herds during 6 consecutive years (Council Directive 64/432/EC). However and despite intensive eradication efforts applied over the years, bTB continues to be present in some other European countries [3]. In Spain, the bTB eradication program has been progressively reinforced through the years (e.g., pre-movement testing, inspections of the field teams, etc), but the OTF status has not yet been achieved. Herd prevalence in 2012 was around 1.3%, but in the last years there has been only a moderate decline [4]. This stagnation is related to the high number of new infected herds detected each year. Between 2006 and 2011, approximately 50% of the positive herds were new infected herds [5], and that poses a serious challenge towards the eradication of the disease.

A bTB herd breakdown may occur due to the persistence of the mycobacteria within the herd (i.e. residual infection), or because of its introduction in a previously free herd. Residual infection could be due to the presence of false negatives to the skin test, reviewed by De la Rua-Domenech [6], or be the consequence of the incorrect application of the test [7]. Also, indirect transmission due to the persistence of the microorganism in the environment could result in residual infections [8]. The presence of infected goats in the farm could also contribute to the recirculation of bTB within the cattle herd [9]–[12].

As external sources of bTB infection, the purchase of infected animals and the interaction with infected cattle or goats at common pastures could be the origin of bTB breakdowns [13]–[16]. The presence of neighboring bTB positive herds may also result in the introduction of the mycobacteria into a herd, via direct contact with infected animals over farm boundaries, or by drainage of contaminated sewage [17]–[20]. In many countries, the presence of wildlife reservoirs endemically infected poses a challenge to bTB eradication schemes. Examples of such reservoirs include the European badger (*Meles meles*) in Great Britain and Ireland [21], [22] or the brushtail possum (*Trichosurus vulpecula*) in New Zealand [19]. In Spain, the Eurasian wild boar (*Sus scrofa*), the red deer (*Cervus elaphus*) and the fallow deer (*Dama dama*) have been identified as bTB maintenance hosts [23], [24], [25]. Finally, humans infected with tuberculosis could also act as a source of infection for cattle [26]–[29].

The determination of the mechanisms by which herds get infected, and the quantification of their relative importance, could be useful information to determine what would be the most appropriate and cost effective preventive measures. Therefore, the main objective of this study was to identify the most likely causes of the bTB herd breakdowns detected in Spain between 2009 and 2011.

## Materials and Methods

### Data

The Spanish national bTB eradication program, according to Council Directive 64/432/EEC, is based on periodical testing of cattle and culling of positive cattle. In each herd test, all animals older than 6 weeks of age are tested annually with the single intradermal test (SIT). Herds are classified as bTB-free if no positive animals are detected in at least two consecutive follow-up herd tests, and as non-bTB free if at least one positive animal is detected. In newly infected herds, based on animal field testing, confirmation of infection is performed by tissue culture for isolation of the causative agent. If the herd is confirmed as infected an epidemiological questionnaire is carried out by a veterinary officer and data is stored in a national database called BRUTUB, which is maintained by the Spanish Ministry of Agriculture, Food and Environment (MAGRAMA) [4]. The questionnaire registers data about management of the herd, history of bTB testing results, animal movements, bTB status of neighbor herds, and interaction with other domestic and wild animals. Besides, the most likely cause of the breakdown in the opinion of the veterinary officer conducting the survey is also recorded. This questionnaire can be accessed in [30]. Data recorded in BRUTUB between 2009 and 2011 were obtained from MAGRAMA.

Additional data about animal movements and bTB status of herds with epidemiological links (i.e., related due to animal movements, neighborhood or pastures) with the studied herds were obtained also from MAGRAMA. For Catalonia (north-eastern Spain), we had access to the ear tag number of all the reactor animals detected in the breakdown, which allowed us to trace individual animal movements. Those data were obtained from the Department of Agriculture, Food and Environment of the Autonomous Government of Catalonia (DAAM).

Also, within the Spanish national bTB eradication program a molecular technique called spoligotyping is applied to strains isolated from the breakdowns. By this technique strains are classified in different groups called spoligotypes as a function of the polymorphism detected within a region in the bacterial genome [31]. The spoligotype patterns of the different isolates of *M. bovis* and *M. caprae* from domestic animals and wildlife (aggregated at municipality level) related with the breakdowns under study were obtained from the mycoDB.es database [32]. The spoligotype patterns of the isolates from the studied herds were provided by the VISAVET Health Surveillance Center located at the Complutense University of Madrid. Additional molecular data from wildlife isolates at county level were provided by the Research Center for Hunting Studies (IREC) and the regional governments of Andalusia and Galicia. Data about bTB testing results in goats were also provided by regional governments.

### Statistical analysis

Descriptive statistics of the number of reactors and within herd incidence by type of production (i.e., beef, dairy or bullfighting) and method of detection (i.e., slaughterhouse, epidemiological link or routine testing) of those breakdowns recorded in the BRUTUB database between 2009 and 2011 were calculated. Differences between groups were assessed by an analysis of variance model and Tukey's test. Due to their highly right skewed distribution the variables were log transformed. The level of significance for the analyses was set to p<0.05. These analyses were performed by using the free software R version 3.0.2.

### Investigation of the most likely cause of bTB herd breakdowns

**Figure 1:**
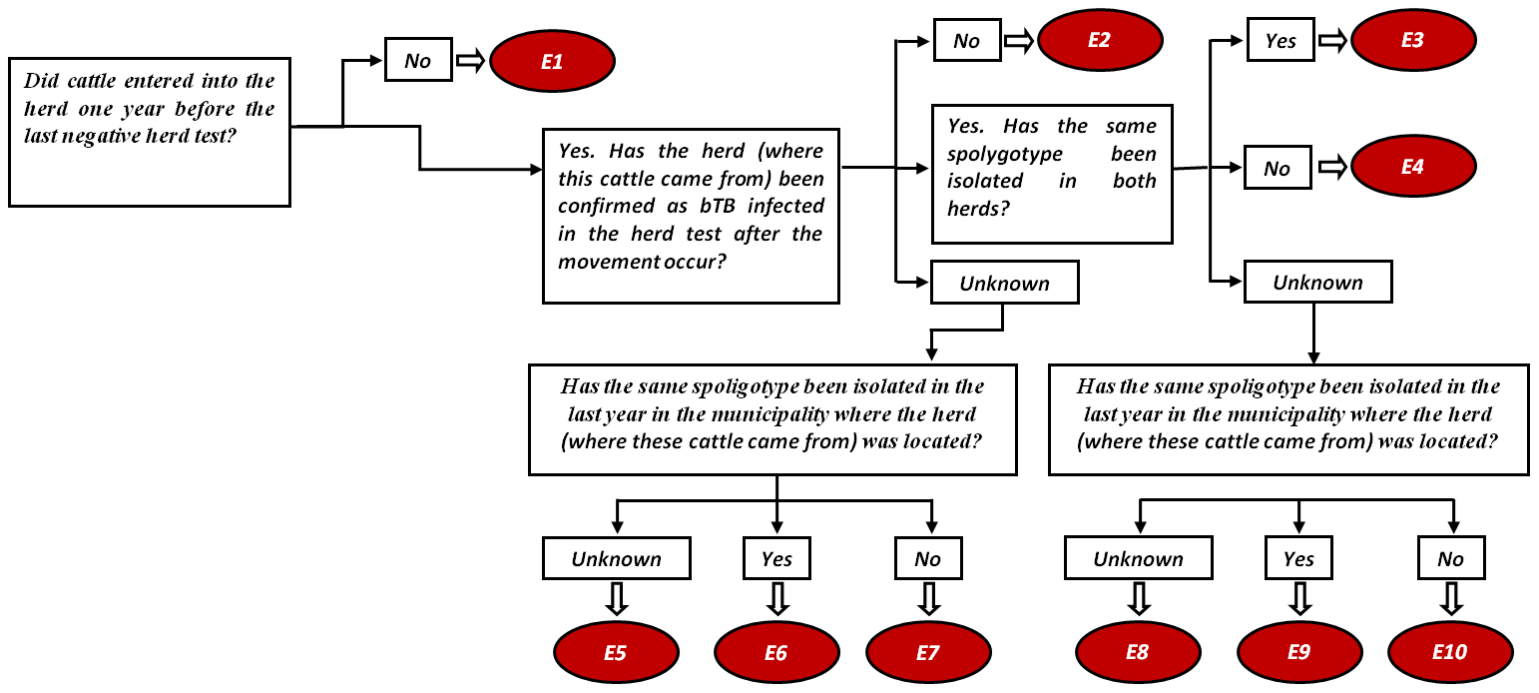
Introduction of infected cattle from other herds decision tree.

In order to assess the most likely cause of bTB breakdowns we followed these steps:

1. Determination of the possible causes of a bTB herd breakdown. Based on bTB epidemiology we considered seven possible causes of herd breakdowns: i) residual infection; ii) introduction of infected cattle from other herds; iii) sharing of pastures with infected herds; iv) contiguous spread from infected neighbor herds; v) presence of infected goats in the farm; vi) interaction with wildlife reservoirs; and vii) contact with an infected human. If the origin of the breakdown could not be attributed to any of the previous causes, it was considered as unknown.
2. Determination of the different events within each possible cause.For each possible cause a decision tree was developed and key questions where included in each of them. Answers to these key questions lead to different events within each decision tree. In figure 1, the decision tree for the introduction of infected animals is shown. The rest of the decision trees are included in the supplementary material (figure S1 in File S1). For example, event E3 in figure 1 would correspond to a herd that had introduced cattle into the herd one year before their last negative herd test. At least one animal came from a herd that had been confirmed as bTB-infected in the herd test after the movement occurred (note that bTB-infected herds are not allowed to move cattle to other herds). Moreover, the same spoligotype was isolated in the herds of origin and destination.
3. Assessment of the likelihood of occurrence of the different events.In order to assess the likelihood of occurrence of the different events a qualitative risk assessment approach was used. For this purpose, an expert opinion workshop was organized following recommendations included in the Handbook on Import Risk Analysis for Animals and Animal Products [33]:
  - We selected experts on the basis of their knowledge, and from a variety of disciplines concerned with the subject. The participants in our Workshop included experts with different backgrounds (i.e., researchers working on domestic and wildlife bTB epidemiology, veterinarians working at regional and central administrations), and came from different regions of Spain (with different epidemiological situations). In order to facilitate the discussion among experts a “manageable number” of experts are recommended. For this workshop nine national experts where contacted. In table S2 in File S1 in the supplementary material a table with the background and expertise of the different national experts that participated in the workshop can be found.
  - Once they agreed to participate, an introduction about expert opinion methodology together with the decision trees was sent to the experts by email, so that they had time to think about it before the meeting. Following recommendations by Dufour et al., [34] ordinal values on the scale of 0 to 9 (table 1) were used.
  - A one day workshop was held in June 2012 in the Veterinary Faculty of the Autonomous University of Barcelona (UAB). In order to solve doubts and avoid misunderstandings, a brief introduction about expert opinion was given together with the instructions on how to assign the values.
  - Time was given to the experts to, individually, assign the considered ordinal values described in table 1 to the different events included in each decision tree.
  - After that, break time was given to the experts, and during that time all results were compiled. Histograms showing the distribution of the ordinal values assigned by experts to each event were prepared.
  - These histograms were discussed with the entire group. During this discussion, experts had the chance to change their ordinal values if they considered that they had overestimated or underestimated any of the events.
  - Finally, descriptive statistics of the nine values provided by the experts in this second questionnaire to each of the 56 events across all decision trees were calculated. The mean value of each of the events was assumed to be the likelihood of occurrence of each event and the mean value of the standard deviations associated with each of them was considered as the overall variability of the experts' opinion. In table S3 in File S1 included in the supplementary material a table with the descriptive statistics of each of the events, a histogram of the standard deviations associated with each of them and a table with the raw values given by the 9 experts in the second questionnaire can be found. Further details related with the “Workshop Method” can be found in the Handbook on Import Risk Analysis for Animals and Animal products [33].
4. Data management and determination of the different events that had occurred in each herd breakdown. Based on available data for each breakdown, we extracted the events, within each possible cause of infection, that had happened following the criteria described in the decision trees (e.g., did cattle enter the herd one year before the last negative herd test?; If yes, has the herd (where these cattle came from) been confirmed as bTB-infected in the herd test after the movement occurred? and so on). Therefore, each herd finished with seven ordinal values (i.e., the likelihood of occurrence of each possible cause of breakdown). In order to perform this task automatically we developed a visual basic macro in Excel. Thanks to this macro, relevant data in the different data files was searched and a new file was generated containing the seven ordinal values by breakdown.
5. Determination of the most likely cause of each bTB herd breakdown.In order to determine the most likely cause of the breakdown for each infected herd, the values of the seven different causes (i.e., the mean ordinal value of each event obtained in the expert opinion workshop) were compared following this criterion:
  - When the seven possible causes of breakdown had values less than 5, the cause of infection of the herd was considered as unknown.
  - In each breakdown, causes for which a value of 5 or more had been assigned were compared among them following these steps:
  -
    - First, we ranked the values from the highest to the lowest value.
    - Then, the cause with the maximum value was considered as the most likely if the difference with the second one was higher than the mean value of the standard deviations of the different events (i.e., one point).
    - In those breakdowns in which three or more causes were within this interval (i.e., three or more values within the highest value minus one point) the cause of infection was considered as unknown.
    - In those breakdowns in which only two causes were within that interval, we considered both options as equally likely, and we assigned 0.5 points to each cause.
    - The 95% confidence intervals of the proportion of each of the most likely causes of breakdown were calculated with the free software R version 3.0.2 using the epiR library [35].

**Table 1:** Ordinal values and categories used for the qualitative risk assessment [34].

Ordinal scaling	Categories
0	Null
1	Nearly null
2	Minute
3	Extremely low
4	Very low
5	Low
6	Not very high
7	Quite high
8	High
9	Very high

### Most likely causes of breakdown attributed by veterinary officers versus causes obtained in our study

The last question that the veterinary officers had to complete in the epidemiological questionnaire [30] was their opinion about the possible cause of the breakdown. They had the option to provide more than one possible cause. In those breakdowns in which two options had been provided, we assigned a value of 0.5 to each of the causes. When the veterinary officers had selected more than two options we considered the cause of breakdown as unknown. In order to calculate the concordance between the opinion of veterinary officers and our results, we made the comparison only for those herds in which a single cause of infection had been obtained by both methods. The agreement between both results was assessed by the Kappa value [36], and calculated with the free software R version 3.0.2 using the epiR library [35]. Kappa values less than 0.2 were considered as indicative of slight agreement, whereas greater than 0.8 would indicate an almost perfect agreement.

## Results

### Descriptive results

**Figure 2:**
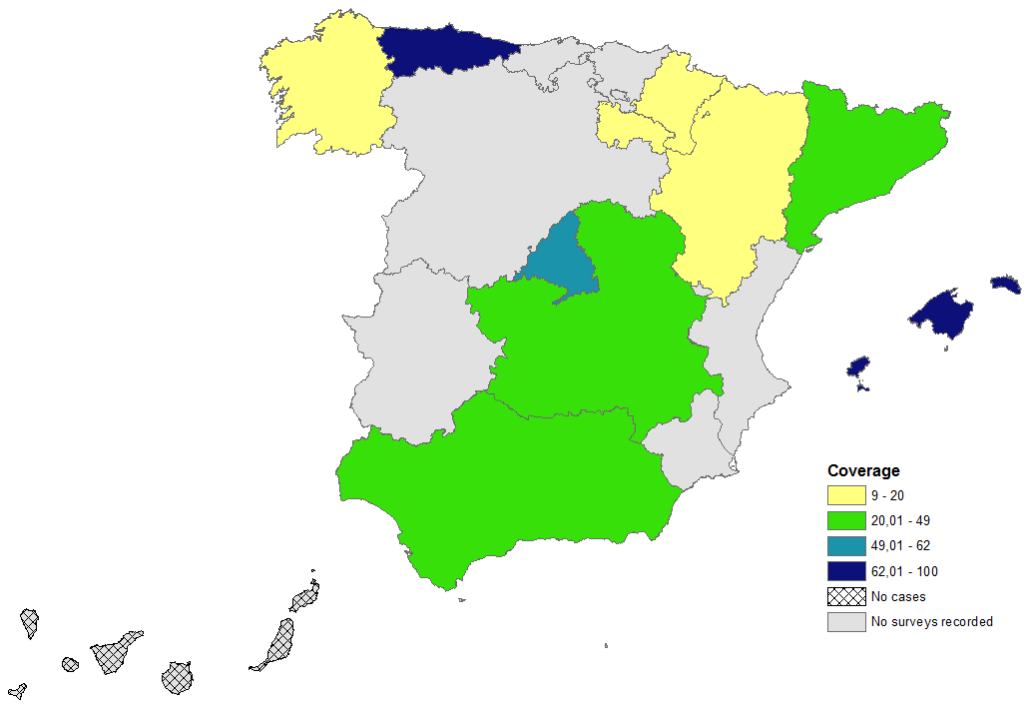
Percentage of breakdowns with a recorded survey (i.e., coverage) between 2009 and 2011.

On 30^th^ May 2012, date when we stopped collecting data, information from 687 breakdowns had been recorded in the BRUTUB system. In figure 2 the geographical distribution of the recorded surveys is represented.

These 687 breakdowns represented the 22% of the breakdowns detected between 2009 and 2011 in Spain. However, the coverage (i.e., percentage of breakdowns recorded in BRUTUB) by regions was variable. There were data of 139 breakdowns from regions with low prevalence (i.e., north and eastern parts of Spain) and of 548 breakdowns from high prevalence regions (i.e., center and south).

Descriptive statistics on the number of reactors and within-herd incidence by type of production (i.e., beef, dairy or bullfighting) and method of detection (i.e., slaughterhouse, epidemiological link or routine testing) are presented in table 2. Bovine TB herd breakdowns were detected mostly by routine herd tests. However, 14% and 22% of breakdowns were detected by slaughterhouse surveillance and epidemiological links (i.e., related by movements, pastures, etc) with infected herds, being an important complement for the detection of the infection.

**Table 2:** Median number of reactors and within herd incidence (in brackets and expressed as a proportion) by detection method (i.e., slaughterhouse surveillance, epidemiological link or routine testing) and herd type (i.e., beef, dairy or bullfighting).

	Beef				Dairy				Bullfighting				Total			
Detection method	Obs	Median	Q3	Max	Obs	Median	Q3	Max	Obs	Median	Q3	Max	Obs	Median	Q3	Max
**Slaughterhouse**	73	3.0(5.3)	9.5(12.5)	128.0(81.5)	18	5.0(3.2)	20.5(15.5)	59.0(63.6)	3	1.0(0.8)	4.0(3.5)	4.0(3.5)	94	3.0(4.3)	9.5(12.3)	128.0(81.5)
**Epidemiological link**	135	4.0(6.5)	7.5(15.9)	65.0(57.4)	6	4.5(6.5)	17.0(26.6)	37.0(86.1)	8	5.0(3.5)	16.0(10.3)	32.0(22.8)	149	4.0(6.4)	7.5(15.6)	65.0(86.1)
**Routine testing**	345	5.0(7.7)	10.0(15.0)	83.0(70.9)	59	2.0(3.2)	5.5(10.0)	110.0(70.9)	26	9.0(6.1)	24.5(10.7)	91.0(49.5)	430	4.0(5.3)	10.0(11.9)	110.0(82.9)
**Total** *	553	4.0(7.1)	9.5(15.5)	128.0(82.9)	83	2.5(3.4)	6.5(10.0)	110.0(86.0)	37	8.0(5.1)	24.0(10.5)	91.0(49.4)	673	4.0(6.6)	9.5(14.5)	128.0(86.0)

*14 farms not included because no data on method of detection recorded.

Obs: number of observed breakdowns; Q3: third quartile; Max: maximum value.

The number of reactors was 4 or lower in half of the breakdowns. Median number of reactors or within herd incidence in herds detected by slaughterhouse surveillance, epidemiological link or routine testing was very similar and no statistically significant differences were identified between them. However, the median within herd incidence was significantly lower on breakdowns detected in dairy (p = 0.007) and bullfighting herds (p = 0.04) compared to beef herds.

### Most likely cause of breakdown based on the decision trees

The most likely causes of herd breakdowns in Spain are shown in table 3. Residual infection was identified as the most important cause (22.3%; 95%CI: 19.4–25.6), followed by interaction with wildlife reservoirs (13.1%; 95%CI: 10.8–15.8). The introduction of infected cattle, sharing of pastures and contiguous spread from infected neighbor herds were also identified as relevant causes. The presence of infected goats and the contact with infected humans seemed to have lower relevance. In 286 herds (41.7%; 95%CI: 38.0–45.4) the origin of infection remained unknown. In 185 of them (64.7%) the likelihood of all the causes was below 5 and in 101 (35.3%) there were more than three plausible causes.

**Table 3:** Most likely causes of bTB breakdowns.

	Most likely		
Causes of breakdown	Herds	Proportion	95% CI
**Residual infection**	153.5	22.3	19.4–25.6
**Introduction of infected cattle**	35	5.1	3.7–7.0
**Presence of infected goats**	17	2.5	1.6–3.9
**Contiguous spread**	55	8	6.2–10.3
**Sharing of pastures**	48.5	7.1	5.4–9.2
**Interaction with wildlife**	90	13.1	10.8–15.8
**Contact with infected humans**	2	0.3	0.1–1.1
**Unknown (a)**	286	41.6	38.0–45.4
**Total**	**687**		

(a) In 185 herds the likelihood of all the causes was below 5 and in 101 there were more than three plausible causes.

95% CI: 95% confidence interval.

If only those herds with a single cause were considered (table 4), residual infection was also the most likely cause followed by interaction with wildlife, contiguous spread and introduction of infected cattle. In this case the importance of sharing of pastures was much lower. In 168 herds, the difference between the first and the second cause with the greater ordinal values was less than one point, for these herds, two possible causes of infection were considered. Within this group, the most frequent first option was residual infection (66.1%), while the most frequent second option was sharing pastures with other herds (48.8%).

**Table 4:** Most likely causes of bTB breakdowns with a single cause (i.e., those breakdowns where the difference between the first and second cause was greater than one point) and with two plausible causes (i.e., herds where the difference between the first and the second cause was less than one point); for these breakdowns we assigned 0.5 points to each cause.

	Most likely		1^st^ most likely		2^nd^ most likely	
Causes of infection	Herds	Proportion	Herds	Proportion	Herds	Proportion
**Residual infection**	83	35.6	111	66.1	30	17.9
**Introduction of infected cattle**	28	12	7	4.2	7	4.2
**Presence of infected goats**	10	4.3	6	3.6	8	4.8
**Contiguous spread**	36	15.5	20	11.9	18	10.7
**Sharing of pastures**	7	3	1	0.6	82	48.8
**Interaction with wildlife**	67	28.8	23	13.7	23	13.7
**Contact with infected humans**	2	0.9	0	0	0	0
***Total***	233		168		168	

There were some differences in the causes of bTB herd breakdown according to the type of herd (table 5). In dairy herds, 65% of the herd breakdowns remained unknown, while wildlife, movements to pastures or contiguous spread seemed to have very little importance. Residual infection was more relevant in bullfighting herds as compared to beef or dairy herds.

**Table 5:** Most likely causes of bTB breakdowns by type of herd.

	Beef	%	Dairy	%	Bullfighting	%
**Residual infection**	126	22.6	15	17.9	11.5	30.3
**Introduction of infected cattle**	22.5	4.0	7	8.3	5	13.2
**Presence of infected goats**	13.5	2.4	2.5	3.0	0	0.0
**Contiguous spread**	49.5	8.9	0.5	0.6	4.5	11.8
**Sharing of pastures**	42	7.5	1.5	1.8	5	13.2
**Interaction with wildlife**	85.5	15.3	0.5	0.6	3	7.9
**Contact with infected humans**	0	0.0	2	2.4	0	0.0
**Unknown**	219	39.2	55	65.5	9	23.7
***Total*** *	558		84		38	

* 7 farms not included (other types).

There were also some differences in the cause of bTB herd breakdowns according to the location of the herd (table 6). In areas of low prevalence such as the north and eastern part of the country, there were a greater percentage of herds with an unknown cause. Contiguous spread and interaction with wildlife reservoirs seemed to have a higher importance in the center and south of the country as compared to the north and eastern areas.

**Table 6:** Most likely causes of bTB breakdowns by area.

	NORTH AND EASTERN	%	CENTER AND SOUTH	%
**Residual infection**	24	17.3	129.5	23.6
**Introduction of infected cattle**	8	5.8	27	4.9
**Presence of infected goats**	2.5	1.8	14.5	2.6
**Contiguous spread**	2	1.4	53	9.7
**Sharing of pastures**	13.5	9.7	35	6.4
**Interaction with wildlife**	11	7.9	79	14.4
**Contact with infected humans**	2	1.4	0	0.0
**Unknown**	76	54.7	210	38.3
***Total***	*139*		*548*	

**Figure 3:**
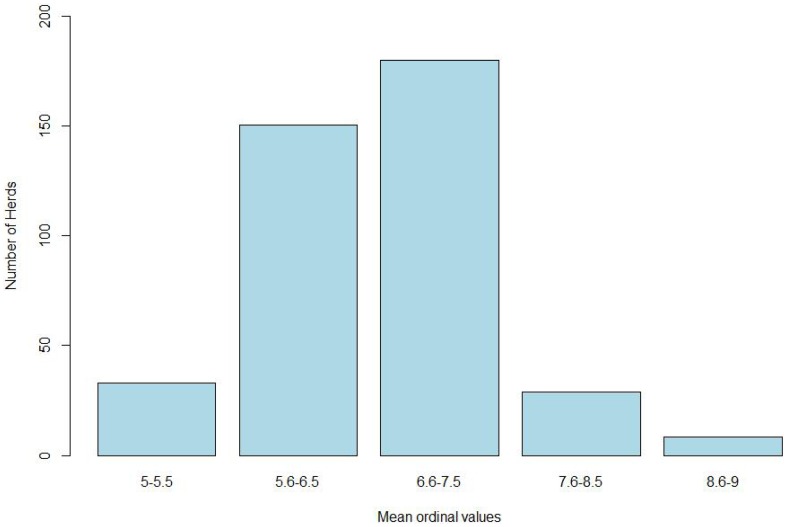
Distribution of the mean ordinal values associated with the most likely cause for each breakdown: “5-.5.5” corresponds to “Low likelihood of occurrence”; “5.6–6.5” to “Not very high likelihood of occurrence”; “6.6–7.5” to “Quite high likelihood of occurrence”; “7.6–8.5” to “High likelihood of occurrence”, and “8.6–9” to a “Very high likelihood of occurrence”.

The mean ordinal values associated with the most likely cause for each breakdown where we could determine a possible cause of the breakdown (i.e., 401 herds) is represented in figure 3. Only in a small proportion of the breakdowns the cause of the breakdown was attributed with a “high” or “very high” likelihood of occurrence. In 29 out of 401 (7%) and in 8.5 out of 401 (2%) of the studied breakdowns the likelihood of occurrence was “high” or “very high” respectively. For the majority of the breakdowns (i.e., 330.5 out of 401 (82%)), the values were between 5.6 and 7.5, which corresponded to qualitative categories of “not very high” and “quite high”. These low values were primarily due to the absence of molecular data, which were lacking for 364 of the 687 studied herds.

In table 7, the most likely events for each cause of infection are represented. Most of the residual infections were attributed to herds that had reactors in the previous 3 years, but for which we did not have enough data to assess whether the isolates had similar molecular characteristics and to herds where the incidence of reactors was not compatible with a recent infection. With regard to the introduction of infected cattle, only a small proportion of the breakdowns (3 out of 35) were associated with a “high” or “very high” likelihood of occurrence. All the breakdowns associated with goats except 1 were due to the presence of goats in the farm, but without data regarding their bTB status. Around 42% of the herds infected by contiguous spread had an infected neighbor herd, but without enough data to assess if they had the same spoligotype. From the breakdowns attributed to wildlife, only in 9.4% the likelihood of occurrence was “high”, and corresponded to herds located near areas of hunting activity and where the spoligotype had been also isolated in wildlife animals of the area.

**Table 7:** The most likely events within each cause of breakdown (see decision trees in figure S1 in File S1 for further clarifications).

Cause of breakdown	Event (value)	Herds	Percentage	Event
**Residual infection**	**E1 (6.1)**	7	4.6	Less than one annual test
	**E2 (7.3)**	56	36.5	Incidence not compatible with a recent infection
	**E4 (5.6)**	2.5	1.6	Reactors in the previous 3 years, but different spoligotype
	**E5 (8.6)**	5.5	3.6	Reactors in previous 3 years and the same spoligotype
	**E6 (6.7)**	82.5	53.7	Reactors in previous 3 years but spoligotype data lacking
	***Total***	153.5		
**Introduction of infected cattle**	**E3 (8.7)**	3	8.6	Herd of origin with the same spoligotype
	**E5 (5.1)**	14.5	41.4	Not known if the herd of origin was positive or if the same spoligotype was present in area of origin
	**E6 (6.4)**	2.5	7.1	Not known if the herd of origin was positive, but the same spoligotype was present in area of origin
	**E8 (6.3)**	12	34.3	Herd of origin was positive, but not known if the same spoligotype was present in area of origin
	**E9 (7.7)**	3	8.6	Herd of origin was positive, and a similar spoligotype was present in area of origin
	***Total***	35		
**Presence of infected goats**	**E4 (6.4)**	16	94.1	Goats present, but bTB status unknown
	**E6 (7.3)**	1	5.9	Positive goats, but spoligotype unknown
	***Total***	17		
**Contiguous spread**	**E2 (7.9)**	16.5	30.0	Positive neighbors and the same spoligotype
	**E3 (5.1)**	1	1.8	Positive neighbors but different spoligotype
	**E4 (5.9)**	23	41.8	Positive neighbors but unknown spoligotype
	**E5 (7.1)**	14.5	26.4	Positive neighbors (with unknown spoligotype) but same spoligotype in the area
	***Total***	55		
**Sharing of pastures**	**E4 (6.3)**	10.5	21.6	With positive herds, but spoligotype unknown
	**E11 (6.0)**	38	78.4	With other herds with unknown bTB status
	***Total***	48.5		
**Interaction with wildlife**	**E2 (5.3)**	4.5	5.0	Unknown if positive wildlife in the area
	**E4 (7.6)**	8.5	9.4	Positive wildlife in the area with the same spoligotype
	**E5 (5.3)**	12	13.3	Positive wildlife in the area, but different spoligotypes
	**E6 (6.2)**	39	43.3	Positive wildlife in the area, but spoligotype unknown
	**E9 (6.4)**	26	28.9	Positive wildlife in the area, with the same spoligotype (but not in hunting area)
	***Total***	90		
**Contact with infected Human**	**E1 (8.4)**	1	50.0	*M.tuberculosis* isolated in the herd, and history of cases in people
	**E3 (5.1)**	1	50.0	*M.tuberculosis* not isolated in the herd, but with history of cases in people
	***Total***	2		

Half values are due to those herds were the difference between the first and the second cause was less than one point. In these breakdowns two possible causes of infection were considered and we assigned 0.5 points to each cause.

### Results of our study versus conclusions from veterinary officers

In 190 breakdowns one single cause was identified as the most likely by both the qualitative assessment and the veterinary officers. Within these herds the agreement between the identified causes of the breakdowns was in general slight (Table 8). The higher disagreement was in the case of introduction of infected cattle and wildlife. Veterinary officers considered that wildlife was the most likely cause for 59 herds, while by applying the decision trees wildlife was linked to only 26 farms, moreover, we just agreed on 12 herds.

**Table 8:** Agreement between causes of breakdown determined by our study and those ones identified by official veterinarians in those herds where we both concluded one option.

	Our study	Veterinary Officer	Agreement	Kappa	IC 95%
**Residual infection**	38	35	12	0.16	0.03–0.31
**Introduction of infected cattle**	13	32	8	0.03	0.00–0.17
**Presence of infected goats**	4	8	0	0	
**Contiguous spread**	9	5	3	0.40	0.27–0.54
**Sharing of pastures**	2	3	2	0.79	0.65–0.93
**Interaction with wildlife**	26	59	12	0.11	0.00–0.23
**Contact with infected humans**	2	1	1	0.39	0.25–0.53
**Unknown**	96	47	38	0.30	0.17–0.42
**Total**	190	190	76		

IC95%: 95% confidence interval for the Kappa statistic.

## Discussion

According to the results of our study, residual infection was identified as the most important cause of bTB breakdowns. This result is in accordance with studies conducted in other European countries where bTB is endemic. In Great Britain, Conlan et al. [37] suggested that up to 21% of herds could harbor the infection after the herd had been classified as bTB free. Moreover, historical bTB incidence has been evidenced as a robust predictor of the rate of future breakdowns in United Kingdom and Ireland [38], [39], [40], [41]. The presence of false negatives animals due to failure of the skin test to detect all the infected animals could be regarded as an important reason to explain the large number of breakdowns attributed to residual infection. However, other factors might be also implicated. In Spain, beef and bullfighting herds are usually kept under extensive conditions in large pasture areas, particularly in Southern and Central regions of the country, which might hinder the testing of all animals [42]. On the other hand, in some breakdowns the incidence found when bTB was first detected at the farm was high (i.e., greater than 25%) which is unusual after a recent infection as bTB is believed to have a low transmission rate within a herd [43], [44], [45]. This could be suggestive of lack of good veterinary practice; however, the presence of other factors that could accelerate bTB transmission, such as the presence of infected males (i.e., could interact with a greater number of cattle and therefore infect a greater number of animals), should not be discarded. The infection appears to be poorly transmitted between cattle in most, but not all circumstances [40]. If this is the case, some of the breakdowns attributed to residual infection could have been misclassified. In addition, the association between previous infection and a breakdown could be not only due to persistence of infected cattle but also to exposure to other risk factors not reflected in the survey (related with lack of biosecurity in high incidence areas), what could induce a certain degree of overestimation of the importance of residual infection.

Herds might also get infected due to an external source. The second most frequent cause of breakdown was the interaction with bTB wildlife reservoirs. In central and southern Spain, high bTB prevalence has been detected in wild boar, red deer and fallow deer, and therefore they could constitute an important source of infection to cattle [23], [24], [25], [46], [47]. In the north of the country the prevalence of infected wildlife reservoirs seems to be lower and therefore their role as bTB reservoirs has been suggested to be of low importance [48]. Moreover, in the northern area there are a higher number of dairy herds with an intensive production system as compared to the central and southern areas of the country. This is to some extent in accordance with the results of our study where wildlife had a higher importance in the central and southern regions of the country. Nevertheless, the evaluation of the role of wildlife was limited by the fact that we did not have data about the presence of bTB in wildlife in the corresponding county for 211 out of 687 studied herds, and for those for which we had data, the molecular identification data were lacking from 260 herds. In 2012, a national surveillance program on bTB in wildlife was launched, and therefore, with the generation of new data, some uncertainty regarding the role of wildlife in different areas of Spain might be clarified.

The importance attributed to the introduction of infected cattle in this study has been lower than that reported in previous ones. In north-east England, Gopal et al. [14] identified the purchase of infected cattle as the most likely source of the infection in 30 of 31 bTB breakdowns. Wilesmith et al. [49] linked the 25% of the breakdowns detected in the period 1972–1978 in Great Britain to animal movements. In Northern Ireland, Denny and Wilesmith [13] based on bTB epidemiological investigations performed by veterinarians from the Department of Agriculture, reported that in 23% of the breakdowns detected in 1996 the source was the purchase of infected cattle. In our opinion, our result is influenced by the quality of the data: in the epidemiological questionnaire only those animal movements considered to pose a risk (i.e. from herds not qualified as officially free for the whole of the last three years) were recorded, and therefore, we did not have data from all the movements. More detailed tracing of animal movements, plus molecular data, would be needed to assess the role of animal movements in bTB breakdowns.

We decided to consider a cause of a herd breakdown only if the likelihood of occurrence was at least “low” (i.e. with a value of 5 in the ordinal scale). This was based on the rationale that those events with a value under 5 corresponded to situations with a negligible biological likelihood of being the cause of the breakdown (e.g. the herd did not have bTB reactors in the previous 3 years together with annual tests conducted each year and an incidence compatible with a recent infection; no cattle have entered into the herd within the date of infection and 1 year before the last negative test, etc). On the basis of this threshold, 27% of the studied herds (i.e. 185 out of 687) were classified as having an unknown cause of breakdown. The rest of “unknown” (i.e. 101 out of 687) corresponded to breakdowns with more than three plausible causes. A 42% of breakdowns with an “unknown” cause of infection are a high number. However, this percentage is in accordance with that reported in other studies from Ireland and Great Britain, where in 32% and 40% of the breakdowns, an infection source could not be established [13], [49]. The determination of the origin of infections, especially in chronic diseases is a difficult task. Moreover, there is not a standard methodology to investigate the cause of a breakdown. Different approaches have been applied in order to determine the possible origin of different diseases; Elbers et al. [50] used key questions to investigate the causes of infection of classical swine fever breakdowns in The Netherlands; the European Food Safety Authority [51] attributed different values to risk factors for bovine cysticercosis by using expert opinion. This methodology was adapted by Allepuz et al. [52] to investigate the most likely causes of infection of bovine cysticercosis in northeastern Spain. The decision trees developed in this study were designed and adapted to get the key information from each possible cause of breakdown. In our view, a key aspect of these decision trees is the assignment of a likelihood of occurrence to each possible event. In order to get estimates as objective as possible we decided to conduct an expert opinion workshop. We tried to reduce the possible bias associated with these estimates by including experts with different backgrounds (i.e. researchers working on domestic and wildlife bTB epidemiology, veterinarians working at regional and central administrations). However, there are inherent limitations derived from obtaining estimates from expert opinion workshops and it would be desirable to repeat this exercise in the future in order to update these values in the light of new scientific evidence about bTB epidemiology and including experts from other regions of Spain.

Moreover, in this study we did not consider some potential causes of infection as the interaction with other potential domestic reservoirs (such as pig or sheep). The role of pigs on bTB epidemiology has been traditionally considered of low importance as they are mainly kept in intensive systems and slaughtered at young ages [53]. However, in the western and southern Spanish regions there is an important population of Iberian breed pigs raised in a free-range system sharing natural resources with other wild and domestic animals. Moreover, in these areas there are reports of Iberian pigs infected with *M. bovis* with generalized lesions [54]. Reports of tuberculosis in sheep have been described in Italy [55] United Kingdom [56] and Spain [57] suggesting their potential to act as a reservoir for tuberculosis. The lack of data from these domestic species, together with the uncertainty regarding their role in bTB epidemiology in Spain made not possible to include them in the analysis. On the other hand, goats were not identified as a relevant cause of bTB breakdowns, which is not in accordance with their potential role in bTB epidemiology [11], [12]. However, it has to be taken into account that just 52 out of the 687 herds reported to have goats in their herd, and only 9 of them had recorded the bTB test results on the survey.

By the development and application of this decision trees, we evaluated different possible causes of bTB breakdowns in the light of available data, and ideally, we should have had enough data in order to discriminate between them. However, for 53% of the breakdowns we did not have molecular data of the mycobacteria isolated in the herd, which limited the evaluation of the different causes, and especially the likelihood to a given cause. Molecular data missing could be due to no collection of the tissue samples at the abattoir, lack of recovery of mycobacteria by culture, typing in progress during the preparation of the manuscript or non-typable collected DNA. The molecular characterization of the different isolates in the breakdowns is essential to provide stronger evidence about the origin of the breakdown.

The comparison carried out between our results and those of the veterinary officers showed a poor agreement. Both methods (decision trees and the opinion of veterinary officers) have weak and strong points, and the reality could be somewhere between the results of both methods. The decision trees are an objective procedure based on expert opinion, group discussion and literature review. Besides, we were able to gather the information later, including some laboratory data that veterinarians might not have had when performing the survey. However, we did not know the particularities of the management, and facilities of each herd and the idiosyncrasy of the area. Besides, the veterinary officers had direct contact with the farm owners to get first hand information. Another likely source of discrepancy between our results and the ones of the veterinary officers is the importance attributed to the different epidemiological contacts. In our study the same criteria was applied to all the herds, while in the case of the veterinary officers there might be a higher heterogeneity due to different regional or individual perceptions about the risk posed by the different epidemiological scenarios. It is remarkable the difference found in the importance attributed to the interaction with wildlife reservoirs. It would be desirable to harmonize the criteria used in the epidemiological investigations conducted by veterinary officers in order to get comparable results between and within the different regions of Spain.

In this study we have analyzed the most likely causes of breakdowns of the 22% of breakdowns detected on different regions of Spain between 2009 and 2011 which corresponds to all the data recorded in the BRUTUB system by 30th May 2011. The unavailability of data from the remaining breakdowns was due to the fact that BRUTUB system was first implemented in 2009 and has been gradually implemented in the different Spanish regions. When interpreting the results, it has to be taken into account that some regions are clearly under-represented and from some regions we did not have data from any breakdown. If there were differences in the causes of breakdowns among regions this would not be reflected in the results of our study. We believe that our results could give a good picture about the most likely causes of bTB herd breakdowns in Spain as we had data from different regions. Nevertheless, it would be desirable to update these analyses in the future when new breakdown data come available.

## Conclusion

Residual infection seems to have an important role as a cause of bTB breakdowns in Spain. This result suggests that focusing efforts in the routine testing procedures in the bTB-positive and recently negative farms should result in an improvement of the eradication program. Nevertheless, it has been evidenced that external sources of bTB had also a relevant role as causes of breakdowns, and therefore measures directed at controlling these factors would be desirable. Interaction with wildlife reservoirs was especially important in the southern parts of the country evidencing that measures to minimize the interaction between infected wildlife reservoirs and domestic animals should contribute to the progress on the eradication of bTB. The high percentage of herds with an “unknown” cause of infection, especially high in areas of low prevalence (i.e., north and eastern parts of Spain), and in dairy herds, reflects the lack of relevant data to infer the most likely cause of breakdown. Gathering more detailed epidemiological information on bTB breakdown investigations together with molecular data would be desirable. The low agreement between the veterinary officer opinion and the results of our study might reflect a lack of harmonized criteria to assess the most likely cause of bTB breakdowns as well as different perceptions about the importance of the possible causes. This is especially relevant in the case of the role of wildlife reservoirs. When interpreting the result it has to be taken into account that a small percentage (i.e. 22%) of the total number of breakdowns detected in Spain between 2009 and 2011 were analyzed in this study, and therefore results have to be interpreted with caution. It would be desirable to update these analyses in the future when new breakdown data become available.

## Supporting Information

File S1 Includes Figures S1–S2 and Tables S1–S4. **Figure S1**. Decision tree diagrams used for the bTB herd breakdowns investigation. **Figure S2.** Histogram of the standard deviations of the different events. **Table S1.** Main data contained in the epidemiological questionnaire carried out by veterinary officers in bTB herd breakdowns. **Table S2.** Background and expertise of the different national experts that participated in the workshop. **Table S3.** Mean ordinal values for each event together with the standard deviation (sd), minimum (min), median and maximum values (max). **Table S4.** Values given by the 9 experts in the expert opinion workshop.
(DOC)
