# Supplementary material for: Epidemiological Investigation of Bovine Tuberculosis Herd Breakdowns in Spain 2009/2011
Source: PLoS One. 2014 Aug 15;9(8):e104383. doi: 10.1371/journal.pone.0104383 (PMC4134210; doi:10.1371/journal.pone.0104383)
Supplement: File S1 — Includes Figures S1–S2 and Tables S1–S4. Figure S1. Decision tree diagrams used for the bTB herd breakdowns investigation. Figure S2. Histogram of the standard deviations of the different events. Table S1. Main data contained in the epidemiological questionnaire carried out by veterinary officers in bTB herd breakdowns. Table S2. Background and expertise of the different national experts that participated in the workshop. Table S3. Mean ordinal values for each event together with the standard deviation (sd), minimum (min), median and maximum values (max). Table S4. Values given by the 9 experts in the expert opinion workshop. (DOC) [file pone.0104383.s001.doc]

- **Table S1: Main data contained in the questionnaire**

| **VARIABLE** | **Description** |
| --- | --- |
| **General data** |  |
| Province | Administrative division |
| County | Administrative division |
| Municipality | Administrative division |
| Previous bTB qualification of the herd | bTB-free ; bTB-non free ; bTB suspicious ; unknwon |
| Date of previous bTB qualification | Date |
| X coordinate | Location |
| Y coordinate | Location |
| Number of cattle | Number |
| Number of sheep | Number |
| Number of goats | Number |
| Type of herd | Dairy, beef, bullfighting ; other |
| Management | Intensive; semi-extensive, extensive |
| Transhumance | If they practice trashumance or not. Transhumance consists on the seasonal movement of animals for feeding purposes. |
| Herd bTB prevalence in the municipality | Number |
| Natural park in the neighbourhood? | Yes; no |
| Neighbor game farm at the neighborhood? | Presence of a game farm (i.e., for hunting activities) |
| Presence of wild boar | If wild boars presence have been detected within the perimeter of the farm. |
| Presence of red deer | If red deer presence have been detected within the perimeter of the farm. |
| Presence of foxes | If foxes presence have been detected within the perimeter of the farm. |
| Presence of badgers | If badgers presence have been detected within the perimeter of the farm. |
| Wildlife can access to the feed of the cattle? | Yes ; no |
| Existence of water points? | Yes ; no |
| Number of water points | Number |
| Wildlife can access the water points? | Yes ; no |
| Do the herd go to pasture? | Yes ; no |
| Shares pasture with other herds? | Yes ; no |
| Can wildlife be present in the pasture? | Yes ; no |
| Hunting residues within farm | In the case hunting activities are performed within the perimeter of the farm, if residues of this activity (i.e., viscera) are normally found. |
| Replacement from positive mothers | If they have done replacement of heifers or males that were born from bTB positive cattle. |
| Cases in people | If there have been cases in people that were working or visiting the farm. |
| Reactors in the last 3 years? | Yes ; no |
| **Herd test data (previous 3 years)** |  |
| Date | Date |
| Reason for conducting the test | Routine testing; epidemiological relation with breakdown; suspicious cattle detected at the slaughterhouse; pre-movement testing |
| Species | Cattle; goats ; other |
| Tested | Number of animals tested |
| Reactors | Number of positive animals to the skin test |
| Percentage of reactors | Number |
| **Laboratory results (previous 3 years)** |  |
| Laboratory test performed? | Yes;no |
| Date | Date |
| Species | Cattle; goats ; other |
| Tested | Number |
| Species | *M. bovis; M. caprae; M. tuberculosis; M. africanum* |
| Test performed | Culture; PCR; spolygotyping; VNTR |
| **Cattle movements** **(previous 3 years)** |  |
| Date of introduction | Date |
| Herd of origin | Code of origin of the movement |
| Number of animals introduced | Number |
| Species | Cattle; goats ; other |
| bTB qualification of the herd of origin | bTB-free ; bTB-non free ; bTB suspicious ; unknwon |
| Has been quarantine performed? | Yes; no |
| Date of pre-movement test on the herd of origin | Date |
| Number of reactors belonging to the movement | Number |
| **Conclusion** |  |
| Conclusion of the veterinary officer | Conclusion of the cause of the breakdown in the opinion of the veterinary officer |

**Figure S1: Decision tree diagrams**

**2.1. Residual infection**

***No. Did the herd conduct at least one annual herd test in the previous3 years?***

***No***

***Is the incidence of reactors compatible with a recent infection (i.e., incidence < 25%)?***

***Did the herd have bTB reactors in the previous 3 years?***

***Yes***

***Yes***

***Yes. Has the same spolygotype been isolated both times?***

***No***

***No***

***Yes***

***Unknown***

*sabe*

**2.2. Introduction of infected cattle from other herds**

***No***

***No***

***Unknown***

***Yes***

***Unknown***

***No***

***Yes. Has the same spolygotype been isolated in both herds?***

***Did cattle entered into the herd one year before the last negative herd test?***

***Yes. Has the herd (where this cattle came from) been confirmed as bTB infected in the herd test after the movement occur?***

***Has the same spoligotype been isolated in the last year in the municipality where the herd (where these cattle came from) was located?***

***Has the same spoligotype been isolated in the last year in the municipality where the herd (where these cattle came from) was located?***

***Unknown***

***Yes***

***No***

***Unknown***

***Yes***

***No***

**2.3. Sharing of pastures with infected herds**

***No***

***Yes. Did the herd shared pastures with a bTB infected herd?***

***Yes***

***Unknown***

***No***

***Has the same spoligotype been isolated in the last year in the municipality where the herd was located?***

***Yes***

***No***

***Unknown***

***Unknown***

***No. Did the herd shared pastures with a goat herd?***

***Does the herd share pastures with other herds or practice transhumance?***

***Yes. Has the same spolygotype been isolated in both herds?***

***No***

***Yes. Has M. caprae been isolated from the herd?***

***Yes***

***No***

***Unknown***

**2.4. Contiguous spread from infected neighbor herds**

***No***

***Yes***

***Unknown***

***No***

***Has the same spoligotype been isolated in the last year in the municipality where the herd was located?***

***Yes***

***No***

***Unknown***

***Is there any bTB infected neighboring farm?***

***Yes. Has the same spolygotype been isolated in both herds?***

**2.5. Presence of infected goats in the farm**

***No***

***Are there goats in the farm?***

***Yes. Have the goats been tested for TB?***

***Yes***

***Unknown***

***No***

***Not or unknown. Has M. caprae been isolated from cattle?***

***Yes. Were the tests positive?***

***Yes***

***No***

***Unknown***

***No***

***Yes. Has the same spolygotype been isolated in cattle and goats?***

**2.6. Interaction with wildlife reservoirs at the farm or pastures**

***Unknown***

***No***

***Yes. Have bTB positive wild animals been found in the region?***

***Yes***

***Unknown***

***No***

***Yes. Has the same spolygotype been isolated in cattle and wild animals?***

***Do red deers, wild boars or badgers have access to feed, water or farm’s pastures?***

***Yes. Is the farm close to a hunting area, or a red deer or wild boar farm?***

***No***

***Unknown***

***No***

***Yes***

***Unknown***

***No***

***No. Have bTB positive wild animals been found in the region?***

***Yes. Has the same spolygotype been isolated in cattle and wild animals?***

**2.7. Contact with an infected human**

***History of cases in people?***

***Yes***

***Has M.tuberculosis been isolated in the farm?***

***No history of cases in people?***

***No***

***History of cases in people?***

***No history of cases in people?***

- **Table S2: Background and expertise of the different national experts that participated in the workshop**

| **Name** | **Institution** | **Background** | **Expertise** |
| --- | --- | --- | --- |
| Mariano Domingo | CReSA | Pathologist | Around 20 years working on bTB research, mainly in domestic animals. |
| Marta Muñoz | Xunta de Galicia | Veterinarian | Around 6 years involved in the planning and coordination of the bTB program in the Department of Rural Affairs of the Regional Government. |
| Irene Mercader | DAAM | Veterinarian | Around 6 years involved in the planning and coordination of the bTB program in the Department of Rural Affairs of the Regional Government. |
| Joaquín Vicente | IREC | Wildlife epidemiologist | Around 8 years working on bTB research, mainly in wildlife animals. |
| Jose Luis Saez | MAGRAMA | Veterinarian | Around 15 years involved in the planning and coordination of the bTB program in the Department of Rural Affairs of the National Government. |
| Julio Álvarez | VISAVET | Diagnostics/epidemiology | Around 10 years working on bTB research, mainly in diagnostic techniques and epidemiology of domestic animals. |
| Bernat Pérez | CReSA | Diagnostics | Around 8 years working on bTB research, mainly with diagnostic techniques. |
| Sebastian Napp | CReSA | Epidemiologist | Around 8 years working on bTB research, mainly with domestic animals. |
| Alberto Allepuz | CReSA | Epidemiologist | Around 6 years working on bTB research, mainly with domestic animals. |

***CReSA:*** Centre de Recerca en Sanitat Animal (located in Barcelona in the north eastern of Spain); ***Xunta de Galicia:*** Department of Rural Affairs of the Regional Government of Galicia (north western Spain); ***DAAM:*** Department of Rural Affairs of the Regional Government of Catalonia (north eastern Spain); ***IREC***: Research Center for Hunting Studies, located in Ciudad Real (southern Spain); ***MAGRAMA:*** Department of Rural Affairs of the National Government; ***VISAVET:*** Health Surveillance Center located at the Complutense University of Madrid (central Spain).

- **Table S3: Mean ordinal values for each event together with the standard deviation (sd), minimum (min), median and maximum values (max).**

| **Cause of breakdown** | **Event** | **mean** | **sd** | **min** | **median** | **max** |
| --- | --- | --- | --- | --- | --- | --- |
| **Residual infection** | E1 | 6.1 | 0.9 | 5 | 6 | 7 |
|  | E2 | 7.3 | 1.1 | 6 | 7 | 9 |
|  | E3 | 3 | 1.3 | 2 | 3 | 6 |
|  | E4 | 5.6 | 2.1 | 2 | 5 | 8 |
|  | E5 | 8.6 | 0.5 | 8 | 9 | 9 |
|  | E6 | 6.7 | 1.3 | 4 | 7 | 9 |
| **Introduction of infected cattle from other farms** | E1 | 0.9 | 1.1 | 0 | 1 | 3 |
|  | E2 | 4.1 | 1.5 | 1 | 5 | 6 |
|  | E3 | 8.7 | 0.7 | 7 | 9 | 9 |
|  | E4 | 5.2 | 1.6 | 2 | 6 | 7 |
|  | E5 | 5.1 | 1.1 | 3 | 5 | 6 |
|  | E6 | 6.4 | 1.2 | 4 | 7 | 8 |
|  | E7 | 4 | 1 | 3 | 4 | 6 |
|  | E8 | 6.3 | 0.7 | 5 | 6 | 7 |
|  | E9 | 7.7 | 0.9 | 7 | 7 | 9 |
|  | E10 | 4.9 | 1.8 | 1 | 6 | 6 |
| **Sharing of pastures with infected herds** | E1 | 0.4 | 0.5 | 0 | 0 | 1 |
|  | E2 | 8.7 | 0.5 | 8 | 9 | 9 |
|  | E3 | 5.8 | 1 | 5 | 5 | 7 |
|  | E4 | 6.3 | 0.7 | 5 | 6 | 7 |
|  | E5 | 7.6 | 0.7 | 7 | 7 | 9 |
|  | E6 | 5.9 | 1.5 | 3 | 6 | 8 |
|  | E7 | 2 | 1.4 | 0 | 2 | 4 |
|  | E8 | 6 | 0.7 | 5 | 6 | 7 |
|  | E9 | 8 | 0.9 | 7 | 8 | 9 |
|  | E10 | 4.4 | 0.9 | 3 | 4 | 6 |
|  | E11 | 6.0 | 0.7 | 5 | 6 | 7 |
| **Contiguous spread from infected neighbor herds** | E1 | 1.8 | 0.7 | 1 | 2 | 3 |
|  | E2 | 7.9 | 0.8 | 7 | 8 | 9 |
|  | E3 | 5.1 | 1.2 | 3 | 6 | 6 |
|  | E4 | 5.9 | 0.6 | 5 | 6 | 7 |
|  | E5 | 7.1 | 0.6 | 6 | 7 | 8 |
|  | E6 | 5.1 | 0.8 | 4 | 5 | 6 |
| **Presence of infected goats in the farm** | E1 | 0.2 | 0.7 | 0 | 0 | 2 |
|  | E2 | 7.4 | 0.7 | 6 | 8 | 8 |
|  | E3 | 6.3 | 0.7 | 5 | 6 | 7 |
|  | E4 | 6.4 | 0.5 | 6 | 6 | 7 |
|  | E5 | 3 | 0.7 | 2 | 3 | 4 |
|  | E6 | 7.3 | 0.7 | 6 | 7 | 8 |
|  | E7 | 8.7 | 0.7 | 7 | 9 | 9 |
|  | E8 | 5.8 | 1.6 | 3 | 6 | 8 |
| **Interaction with wildlife reservoirs at the farm or pastures** | E1 | 1.3 | 0.7 | 0 | 1 | 2 |
|  | E2 | 5.3 | 0.9 | 4 | 5 | 7 |
|  | E3 | 3.6 | 0.9 | 2 | 4 | 5 |
|  | E4 | 7.6 | 0.5 | 7 | 8 | 8 |
|  | E5 | 5.3 | 1.2 | 4 | 5 | 7 |
|  | E6 | 6.2 | 0.8 | 5 | 6 | 7 |
|  | E7 | 4.2 | 1 | 3 | 4 | 6 |
|  | E8 | 3 | 1.5 | 1 | 3 | 6 |
|  | E9 | 6.4 | 0.9 | 5 | 6 | 8 |
|  | E10 | 4.3 | 1.4 | 2 | 5 | 6 |
|  | E11 | 4.9 | 1.3 | 2 | 5 | 6 |
| **Contact with an infected human** | E1 | 8.4 | 0.7 | 7 | 9 | 9 |
|  | E2 | 7.2 | 1 | 6 | 7 | 9 |
|  | E3 | 5.1 | 0.9 | 3 | 5 | 6 |
|  | E4 | 1.3 | 1.1 | 0 | 1 | 3 |

**
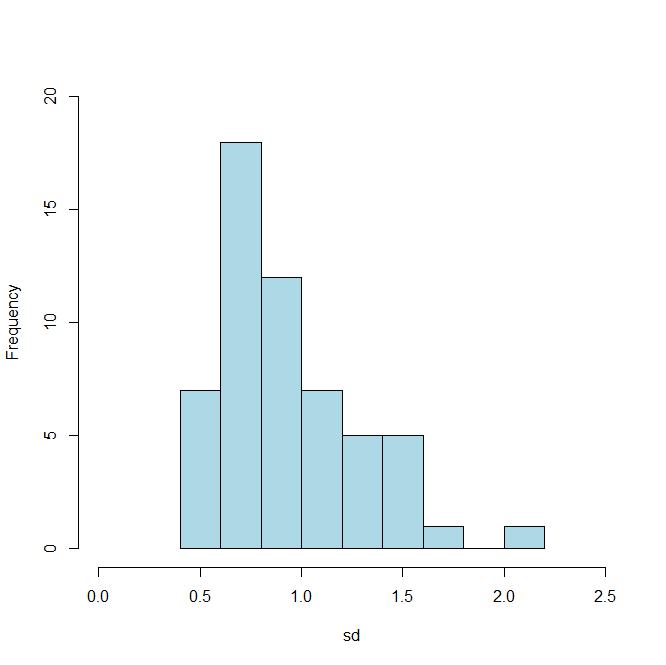
**

- **Figure S2: Histogram of the standard deviations of the different events.**
- **Table S4: Values given by the 9 experts in the expert opinion workshop.**

| **Cause of breakdown** | **Event** | **Value given by expert** |
| --- | --- | --- |
| **Residual infection** | E1 | 7 |
|  | E1 | 7 |
|  | E1 | 7 |
|  | E1 | 6 |
|  | E1 | 6 |
|  | E1 | 5 |
|  | E1 | 5 |
|  | E1 | 5 |
|  | E1 | 7 |
|  | E2 | 6 |
|  | E2 | 9 |
|  | E2 | 7 |
|  | E2 | 8 |
|  | E2 | 6 |
|  | E2 | 7 |
|  | E2 | 7 |
|  | E2 | 7 |
|  | E2 | 9 |
|  | E3 | 2 |
|  | E3 | 6 |
|  | E3 | 4 |
|  | E3 | 3 |
|  | E3 | 3 |
|  | E3 | 2 |
|  | E3 | 2 |
|  | E3 | 2 |
|  | E3 | 3 |
|  | E4 | 5 |
|  | E4 | 8 |
|  | E4 | 7 |
|  | E4 | 4 |
|  | E4 | 7 |
|  | E4 | 5 |
|  | E4 | 2 |
|  | E4 | 4 |
|  | E4 | 8 |
|  | E5 | 9 |
|  | E5 | 9 |
|  | E5 | 9 |
|  | E5 | 8 |
|  | E5 | 9 |
|  | E5 | 9 |
|  | E5 | 8 |
|  | E5 | 8 |
|  | E5 | 8 |
|  | E6 | 6 |
|  | E6 | 9 |
|  | E6 | 7 |
|  | E6 | 4 |
|  | E6 | 7 |
|  | E6 | 7 |
|  | E6 | 7 |
|  | E6 | 6 |
|  | E6 | 7 |
| **Introduction of infected animals from other farms** | E1 | 2 |
|  | E1 | 0 |
|  | E1 | 0 |
|  | E1 | 3 |
|  | E1 | 0 |
|  | E1 | 0 |
|  | E1 | 1 |
|  | E1 | 1 |
|  | E1 | 1 |
|  | E2 | 4 |
|  | E2 | 6 |
|  | E2 | 5 |
|  | E2 | 5 |
|  | E2 | 1 |
|  | E2 | 3 |
|  | E2 | 5 |
|  | E2 | 3 |
|  | E2 | 5 |
|  | E3 | 9 |
|  | E3 | 9 |
|  | E3 | 9 |
|  | E3 | 7 |
|  | E3 | 8 |
|  | E3 | 9 |
|  | E3 | 9 |
|  | E3 | 9 |
|  | E3 | 9 |
|  | E4 | 6 |
|  | E4 | 7 |
|  | E4 | 7 |
|  | E4 | 6 |
|  | E4 | 5 |
|  | E4 | 6 |
|  | E4 | 2 |
|  | E4 | 4 |
|  | E4 | 4 |
|  | E5 | 5 |
|  | E5 | 6 |
|  | E5 | 6 |
|  | E5 | 5 |
|  | E5 | 3 |
|  | E5 | 6 |
|  | E5 | 5 |
|  | E5 | 4 |
|  | E5 | 6 |
|  | E6 | 7 |
|  | E6 | 8 |
|  | E6 | 7 |
|  | E6 | 7 |
|  | E6 | 4 |
|  | E6 | 7 |
|  | E6 | 6 |
|  | E6 | 5 |
|  | E6 | 7 |
|  | E7 | 4 |
|  | E7 | 6 |
|  | E7 | 4 |
|  | E7 | 5 |
|  | E7 | 3 |
|  | E7 | 3 |
|  | E7 | 4 |
|  | E7 | 3 |
|  | E7 | 4 |
|  | E8 | 7 |
|  | E8 | 7 |
|  | E8 | 7 |
|  | E8 | 5 |
|  | E8 | 6 |
|  | E8 | 6 |
|  | E8 | 6 |
|  | E8 | 7 |
|  | E8 | 6 |
|  | E9 | 9 |
|  | E9 | 9 |
|  | E9 | 8 |
|  | E9 | 7 |
|  | E9 | 7 |
|  | E9 | 7 |
|  | E9 | 7 |
|  | E9 | 8 |
|  | E9 | 7 |
|  | E10 | 6 |
|  | E10 | 6 |
|  | E10 | 6 |
|  | E10 | 5 |
|  | E10 | 6 |
|  | E10 | 1 |
|  | E10 | 5 |
|  | E10 | 6 |
|  | E10 | 3 |
| **Sharing of pastures with infected herds** | E1 | 1 |
|  | E1 | 1 |
|  | E1 | 1 |
|  | E1 | 0 |
|  | E1 | 0 |
|  | E1 | 0 |
|  | E1 | 1 |
|  | E1 | 0 |
|  | E1 | 0 |
|  | E2 | 9 |
|  | E2 | 9 |
|  | E2 | 9 |
|  | E2 | 8 |
|  | E2 | 9 |
|  | E2 | 9 |
|  | E2 | 8 |
|  | E2 | 9 |
|  | E2 | 8 |
|  | E3 | 6 |
|  | E3 | 7 |
|  | E3 | 5 |
|  | E3 | 7 |
|  | E3 | 7 |
|  | E3 | 5 |
|  | E3 | 5 |
|  | E3 | 5 |
|  | E3 | 5 |
|  | E4 | 7 |
|  | E4 | 7 |
|  | E4 | 6 |
|  | E4 | 7 |
|  | E4 | 6 |
|  | E4 | 5 |
|  | E4 | 6 |
|  | E4 | 7 |
|  | E4 | 6 |
|  | E5 | 8 |
|  | E5 | 9 |
|  | E5 | 8 |
|  | E5 | 7 |
|  | E5 | 7 |
|  | E5 | 7 |
|  | E5 | 7 |
|  | E5 | 8 |
|  | E5 | 7 |
|  | E6 | 7 |
|  | E6 | 6 |
|  | E6 | 6 |
|  | E6 | 8 |
|  | E6 | 6 |
|  | E6 | 5 |
|  | E6 | 5 |
|  | E6 | 7 |
|  | E6 | 3 |
|  | E7 | 4 |
|  | E7 | 1 |
|  | E7 | 2 |
|  | E7 | 3 |
|  | E7 | 0 |
|  | E7 | 3 |
|  | E7 | 3 |
|  | E7 | 0 |
|  | E7 | 2 |
|  | E8 | 6 |
|  | E8 | 6 |
|  | E8 | 6 |
|  | E8 | 7 |
|  | E8 | 5 |
|  | E8 | 6 |
|  | E8 | 7 |
|  | E8 | 5 |
|  | E8 | 6 |
|  | E9 | 7 |
|  | E9 | 9 |
|  | E9 | 8 |
|  | E9 | 8 |
|  | E9 | 9 |
|  | E9 | 8 |
|  | E9 | 9 |
|  | E9 | 7 |
|  | E9 | 7 |
|  | E10 | 5 |
|  | E10 | 6 |
|  | E10 | 5 |
|  | E10 | 3 |
|  | E10 | 4 |
|  | E10 | 4 |
|  | E10 | 4 |
|  | E10 | 4 |
|  | E10 | 5 |
|  | E11 | 5 |
|  | E11 | 5 |
|  | E11 | 7 |
|  | E11 | 6 |
|  | E11 | 7 |
|  | E11 | 6 |
|  | E11 | 6 |
|  | E11 | 6 |
|  | E11 | 6 |
| **Contiguous spread from infected neighbor herds** | E1 | 2 |
|  | E1 | 2 |
|  | E1 | 1 |
|  | E1 | 2 |
|  | E1 | 1 |
|  | E1 | 2 |
|  | E1 | 2 |
|  | E1 | 3 |
|  | E1 | 1 |
|  | E2 | 9 |
|  | E2 | 7 |
|  | E2 | 9 |
|  | E2 | 7 |
|  | E2 | 7 |
|  | E2 | 8 |
|  | E2 | 8 |
|  | E2 | 8 |
|  | E2 | 8 |
|  | E3 | 6 |
|  | E3 | 6 |
|  | E3 | 6 |
|  | E3 | 6 |
|  | E3 | 5 |
|  | E3 | 6 |
|  | E3 | 4 |
|  | E3 | 4 |
|  | E3 | 3 |
|  | E4 | 6 |
|  | E4 | 6 |
|  | E4 | 6 |
|  | E4 | 7 |
|  | E4 | 5 |
|  | E4 | 6 |
|  | E4 | 5 |
|  | E4 | 6 |
|  | E4 | 6 |
|  | E5 | 8 |
|  | E5 | 7 |
|  | E5 | 8 |
|  | E5 | 7 |
|  | E5 | 6 |
|  | E5 | 7 |
|  | E5 | 7 |
|  | E5 | 7 |
|  | E5 | 7 |
|  | E6 | 6 |
|  | E6 | 6 |
|  | E6 | 5 |
|  | E6 | 6 |
|  | E6 | 5 |
|  | E6 | 4 |
|  | E6 | 4 |
|  | E6 | 5 |
|  | E6 | 5 |
| **Presence of infected goats in the farm** | E1 | 0 |
|  | E1 | 0 |
|  | E1 | 0 |
|  | E1 | 2 |
|  | E1 | 0 |
|  | E1 | 0 |
|  | E1 | 0 |
|  | E1 | 0 |
|  | E1 | 0 |
|  | E2 | 8 |
|  | E2 | 8 |
|  | E2 | 8 |
|  | E2 | 6 |
|  | E2 | 7 |
|  | E2 | 7 |
|  | E2 | 8 |
|  | E2 | 8 |
|  | E2 | 7 |
|  | E3 | 6 |
|  | E3 | 7 |
|  | E3 | 5 |
|  | E3 | 7 |
|  | E3 | 6 |
|  | E3 | 6 |
|  | E3 | 7 |
|  | E3 | 6 |
|  | E3 | 7 |
|  | E4 | 6 |
|  | E4 | 7 |
|  | E4 | 6 |
|  | E4 | 6 |
|  | E4 | 6 |
|  | E4 | 6 |
|  | E4 | 7 |
|  | E4 | 7 |
|  | E4 | 7 |
|  | E5 | 3 |
|  | E5 | 3 |
|  | E5 | 4 |
|  | E5 | 2 |
|  | E5 | 2 |
|  | E5 | 4 |
|  | E5 | 3 |
|  | E5 | 3 |
|  | E5 | 3 |
|  | E6 | 7 |
|  | E6 | 8 |
|  | E6 | 7 |
|  | E6 | 7 |
|  | E6 | 8 |
|  | E6 | 7 |
|  | E6 | 8 |
|  | E6 | 6 |
|  | E6 | 8 |
|  | E7 | 9 |
|  | E7 | 9 |
|  | E7 | 9 |
|  | E7 | 7 |
|  | E7 | 9 |
|  | E7 | 9 |
|  | E7 | 9 |
|  | E7 | 8 |
|  | E7 | 9 |
|  | E8 | 4 |
|  | E8 | 7 |
|  | E8 | 6 |
|  | E8 | 6 |
|  | E8 | 8 |
|  | E8 | 6 |
|  | E8 | 5 |
|  | E8 | 3 |
|  | E8 | 7 |
| **Interaction with wildlife reservoirs at the farm or pastures** | E1 | 2 |
|  | E1 | 0 |
|  | E1 | 1 |
|  | E1 | 2 |
|  | E1 | 1 |
|  | E1 | 2 |
|  | E1 | 2 |
|  | E1 | 1 |
|  | E1 | 1 |
|  | E2 | 6 |
|  | E2 | 5 |
|  | E2 | 5 |
|  | E2 | 7 |
|  | E2 | 4 |
|  | E2 | 5 |
|  | E2 | 5 |
|  | E2 | 6 |
|  | E2 | 5 |
|  | E3 | 4 |
|  | E3 | 3 |
|  | E3 | 4 |
|  | E3 | 3 |
|  | E3 | 2 |
|  | E3 | 3 |
|  | E3 | 4 |
|  | E3 | 5 |
|  | E3 | 4 |
|  | E4 | 8 |
|  | E4 | 7 |
|  | E4 | 7 |
|  | E4 | 8 |
|  | E4 | 8 |
|  | E4 | 7 |
|  | E4 | 8 |
|  | E4 | 8 |
|  | E4 | 7 |
|  | E5 | 5 |
|  | E5 | 6 |
|  | E5 | 4 |
|  | E5 | 7 |
|  | E5 | 7 |
|  | E5 | 5 |
|  | E5 | 4 |
|  | E5 | 6 |
|  | E5 | 4 |
|  | E6 | 7 |
|  | E6 | 6 |
|  | E6 | 5 |
|  | E6 | 7 |
|  | E6 | 7 |
|  | E6 | 5 |
|  | E6 | 6 |
|  | E6 | 6 |
|  | E6 | 7 |
|  | E7 | 5 |
|  | E7 | 4 |
|  | E7 | 3 |
|  | E7 | 6 |
|  | E7 | 4 |
|  | E7 | 4 |
|  | E7 | 4 |
|  | E7 | 5 |
|  | E7 | 3 |
|  | E8 | 3 |
|  | E8 | 1 |
|  | E8 | 2 |
|  | E8 | 6 |
|  | E8 | 4 |
|  | E8 | 2 |
|  | E8 | 3 |
|  | E8 | 4 |
|  | E8 | 2 |
|  | E9 | 8 |
|  | E9 | 6 |
|  | E9 | 6 |
|  | E9 | 7 |
|  | E9 | 6 |
|  | E9 | 6 |
|  | E9 | 7 |
|  | E9 | 7 |
|  | E9 | 5 |
|  | E10 | 5 |
|  | E10 | 5 |
|  | E10 | 3 |
|  | E10 | 6 |
|  | E10 | 6 |
|  | E10 | 3 |
|  | E10 | 4 |
|  | E10 | 5 |
|  | E10 | 2 |
|  | E11 | 6 |
|  | E11 | 5 |
|  | E11 | 2 |
|  | E11 | 6 |
|  | E11 | 6 |
|  | E11 | 4 |
|  | E11 | 5 |
|  | E11 | 5 |
|  | E11 | 5 |
| **Contact with an infected human** | E1 | 9 |
|  | E1 | 9 |
|  | E1 | 9 |
|  | E1 | 8 |
|  | E1 | 9 |
|  | E1 | 8 |
|  | E1 | 9 |
|  | E1 | 8 |
|  | E1 | 7 |
|  | E2 | 6 |
|  | E2 | 9 |
|  | E2 | 8 |
|  | E2 | 7 |
|  | E2 | 8 |
|  | E2 | 7 |
|  | E2 | 7 |
|  | E2 | 7 |
|  | E2 | 6 |
|  | E3 | 6 |
|  | E3 | 5 |
|  | E3 | 5 |
|  | E3 | 5 |
|  | E3 | 6 |
|  | E3 | 5 |
|  | E3 | 3 |
|  | E3 | 5 |
|  | E3 | 6 |
|  | E4 | 3 |
|  | E4 | 0 |
|  | E4 | 3 |
|  | E4 | 0 |
|  | E4 | 1 |
|  | E4 | 1 |
|  | E4 | 1 |
|  | E4 | 1 |
|  | E4 | 2 |
